# Mating Reverses Actuarial Aging in Female Queensland Fruit Flies

**DOI:** 10.1371/journal.pone.0132486

**Published:** 2015-07-06

**Authors:** Sarsha Yap, Benjamin G. Fanson, Phillip W. Taylor

**Affiliations:** Department of Biological Sciences, Macquarie University, Sydney, NSW 2109, Australia; University of Arkansas, UNITED STATES

## Abstract

Animals that have a long pre-reproductive adult stage often employ mechanisms that minimize aging over this period in order to preserve reproductive lifespan. In a remarkable exception, one tephritid fruit fly exhibits substantial pre-reproductive aging but then mitigates this aging during a diet-dependent transition to the reproductive stage, after which life expectancy matches that of newly emerged flies. Here, we ascertain the role of nutrients, sexual maturation and mating in mitigation of previous aging in female Queensland fruit flies. Flies were provided one of three diets: ‘sugar’, ‘essential’, or ‘yeast-sugar’. Essential diet contained sugar and micronutrients found in yeast but lacked maturation-enabling protein. At days 20 and 30, a subset of flies on the sugar diet were switched to essential or yeast-sugar diet, and some yeast-sugar fed flies were mated 10 days later. Complete mitigation of actuarial aging was only observed in flies that were switched to a yeast-sugar diet and mated, indicating that mating is key. Identifying the physiological processes associated with mating promise novel insights into repair mechanisms for aging.

## Introduction

Selection pressures on aging are usually strongest prior to reproduction, because at this time the full extent of reproductive potential remains at stake. To preserve reproductive lifespan, animals are expected to have mechanisms that minimize aging during pre-reproductive stages. In insects the soma is mitotically active throughout the pre-adult stages, and is hence capable of repair and renewal. As a consequence, insects can reach the adult life stage with little accumulated somatic damage [1, 2]. Once the adult stage is reached, however, the soma is mainly post-mitotic, resulting in the accumulation of somatic damage and hence aging [1, 3]. Insects that reproduce soon after reaching the adult stage are exposed to minimal fitness costs of cumulative somatic damage as their reproductive potential may be largely fulfilled before being constrained by aging. However, some insects require substantial time during the adult stage to complete sexual development (e.g. mosquito [4]; blowfly [5]; lubber grasshopper [6]), resulting in a sometimes lengthy period of susceptibility to pre-reproductive aging. Consequently, insects have evolved several mechanisms that enable them to traverse pre-reproductive adult stages and enter their reproductive stage with minimal accrual of somatic damage and aging.

The most common mechanism for minimizing aging prior to sexual reproduction entails prevention; many insects are able to minimize pre-reproductive aging by entering reproductive diapause, during which protective mechanisms are up-regulated and aging is largely held at bay (Panel a in S1 Fig; reviewed in [1]). A far less known mechanism instead entails recovery. Mitigation of pre-reproductive adult aging has been shown for only one insect species to date: the Mediterranean fruit fly (‘medfly’; *Ceratitis capitata*) [7] which require a protein meal to complete sexual maturation (i.e., ‘anautogenous’). When adult female medflies were maintained on a sugar-only diet from emergence, they remained reproductively immature and expressed the increasing mortality rate characteristic of aging. When they were later provided yeast – a source of the protein required for sexual maturation – and allowed to mate at day 30, 60, or 90 post-emergence, mortality rate dropped to zero and thereafter matched that of newly emerged flies (Panel b in S1 Fig) [7]. These results suggest activation of repair mechanisms that mitigated aging during transition to the reproductive adult stage.

Nutrition has been assumed to be directly responsible for the mitigation of aging when medflies transition to the reproductive mode of aging [7–9]. Nutrition can affect mortality rates by changing both acute mortality risk and aging rates [10, 11], but there is no clear empirical evidence to support the idea that nutrition alone can mitigate previous aging. On the other hand, the possibility that diet-associated ontogenic processes (e.g. sexual maturation) mediate this aging pattern have been neglected. Recent research with honeybees (*Apis mellifera*) points to involvement of ontogenetic processes in mitigation of aging, as bees switched from the foraging stage back to the nursing stage expressed mitigation of previous aging (reviewed in [12, 13]).

Mating state can also influence aging, reducing mortality rate in some cases [14, 15] and increasing mortality rate in others [15, 16], but the possibility that mitigation of aging reported in medflies is related to mating that occurred as a consequence of sexual maturation enabled by diet has not been considered. It is hence unclear whether mitigation of pre-reproductive aging in medflies is mediated directly by nutrition or is instead mediated by diet-associated ontogenic processes or subsequent mating. The present study takes an essential next step toward understanding the mechanisms by which aging is mitigated during the transition to reproductive mode in tephritid fruit flies. Here, we ascertain the roles of nutrition, ontogeny and mating as triggers for mitigation of aging in the Queensland fruit fly (*Bactrocera tryoni*, ‘Q-fly’), an anautogenous tephritid fly with pre-reproductive aging patterns that closely resemble those of medflies [17, 18].

## Materials and Methods

### Fly stock and housing

Queensland fruit flies were obtained as pupae from a mass-reared population at Elizabeth Macarthur Agricultural Institute, New South Wales, Australia. Within 48 h of emergence, female Q-flies were separated and housed individually in 70 mL clear plastic specimen containers with six holes (2 mm in diameter) drilled through the bottom surface for ventilation. Each container was placed upside down on an ovipositing substrate, which comprised the container lid filled with 0.7% lemon essence solution (Queens Fine Foods Pty Ltd) and covered with parafilm that had been pierced four times with an entomological pin. Flies oviposited through the parafilm and eggs dropped into the water below. Experimental diets were placed on the parafilm and deionised water was provided through a 200 μL pipette tip inserted through one of the ventilation holes. The ovipositing substrate and food were replaced every 5 days. Flies were maintained at 25–26°C and 80–85% humidity with a photoperiod of 13L:11D in which the light phase included a simulated daily dawn and dusk during which light levels ramped up and down, respectively, over 1 h.

### Diets

We created three agar-based experimental diets: ‘sugar’ (SUG), ‘yeast-sugar’ (YS) and ‘essential’ (ESS) (S1 Table). The SUG diet contained sucrose, agar, nipagin and deionised water. For the YS diet, we combined hydrolysed yeast with the SUG diet in a 1:3 yeast-sucrose ratio. For the ESS diet, we added Vanderzant vitamin mixture, Wesson salt mixture and cholesterol to the SUG diet. ESS diet provides essential nutrients found in hydrolysed yeast but differs from YS diet in lacking reproduction-triggering protein. All diets had a concentration of 300 g/L and were prepared by measuring the ingredients by weight and combining them with deionised water. Agar was prepared separately by boiling in deionised water. The diet components and agar were then mixed to a uniform consistency, and allowed to set in a 75 mL cubic plastic container in a refrigerator. Once set, the diet was removed from the container as a single block. The diet block was then cut into cubes of approximately 4 mm^3^. Fresh blocks of diets were prepared every week.

### Experimental protocol

The experimental protocol used with medflies [7] involved switching female flies from a sugar diet to a yeast-sugar diet while paired with a male. As a result, when female flies were switched to a yeast-sugar diet, a series of interventions occurred in concert: (1) nutrient-deprived flies acquired protein and micronutrients (e.g., vitamins, minerals, sterols); (2) protein combined with other nutrients enabled reproductive maturation; and (3) female flies mated, receiving sperm and seminal fluids. Any one (or a combination) of these events may be responsible for mitigating accrued aging. To better characterize each component’s role, we modified the experimental protocol used with medflies [7].

Here, we switched flies from the SUG diet to either the YS or ESS diet at day 0, 20 and 30 after emergence (S2 Fig). Within 48 hours of emergence, we provided 1518 Q-flies with SUG diet (SUG-0) and deionised water. Another 400 Q-flies were allocated to either the ESS diet (ESS-0) or the YS diet (YS-0-U) at this time. At day 20, a subset of Q-flies from the SUG diet was switched to the YS diet (‘YS-20-U’, N = 100) or the essential diet (‘ESS-20’, N = 50). Finally, at day 30, a subset of Q-flies was switched from the SUG diet to the YS diet (‘YS-30-U’, N = 80) or the ESS diet (‘ESS-30’, N = 30). For Q-flies placed on the YS diet from days 0, 20 and 30 a sample of 97 (YS-0-M), 37 (YS-20-M) and 22 (YS-30-M) flies respectively were given access to mates 10 days after being switched to the diet in order to give females time to develop their reproductive organs. Ovipositing dishes were provided from day 10 onwards and were replaced every 5 days. Egg production and survival were monitored until the last female died. Mortality was checked daily.

### Mating trials

Q-flies only mate at dusk and our pilot studies indicated that mating is more likely when multiple males are present. For each female to be mated, two males that had been fed YS diet for 10–14 days were introduced to each female’s container two hours before dusk. At this age, male Q-flies maintained on this diet are at peak mating activity [19]. Copulations were observed during dusk, and those females that copulated were deemed ‘mated’. Females that did not mate during the first trial were given a second opportunity to mate the next day with new males. After the second mating opportunity, females that did not copulate were excluded. All males were removed from the containers the morning after the mating trials.

### Dissections

To determine the effects of diet on ovarian development (reproductive maturation) and to confirm inhibition of reproductive maturation for flies receiving SUG and ESS diets, Q-flies from each treatment were dissected after they had died. For each dissection, images of the ovaries were captured with a camera (Progres C10, 3 MegaPixels) attached to the phototube of an Olympus SZX12 dissecting microscope and the ovaries were then examined from these images. Ovarian development was assessed according to: (a) the stage of development, scored on a scale developed by [20] (1 and 2 representing previtellogenesis, 3 and 4 vitellogenesis and 5 mature egg formation), and (b) whether eggs were present in the ovaries.

### Statistical analyses

All statistical analyses were conducted using SAS 9.1. To assess the effect of experimental diets, switches between diets, and mating on mortality rate patterns, we first compared remaining life expectancies using general linear models at key time points (0, 10, 20, and 30 days) among diet and mating treatments. Expected remaining life expectancies were computed as the number of days remaining for the average individual alive at day X. Additionally, we performed nonparametric survival analyses to compare mortality trajectories, using Wilcoxon-Gehan tests for treatment comparisons. Wilcoxon-Gehan tests are preferred over log-rank tests when survival curves do not meet the proportional hazard (PH) assumption, such as was the case for our comparisons of survival curves between diets treatments. Further, Wilcoxon-Gehan tests emphasise observations closer to the switch points, which are the focus of our hypotheses [21]. For graphical depictions of mortality trajectories, we fitted a weighted LOESS curve (span = 0.4 for all curves) to daily mortality rates for each treatment group in R (v2.9.0). Separately for the mean lifespan comparisons and the survival curve comparisons, we performed a false discovery rate correction to the p-values to adjust for multiple comparisons.

To assess the effects of diet on egg production rates, we first used a logistic regression model to examine the effects of switch day and mating status on the probability of laying any eggs. Next we examined individual egg production rates over time for those flies that laid eggs using a general linear mixed model. In this analysis, mating status, switching time and each 5-day interval were fixed variables and female identity was included as a random factor. Given mating occurred 10–11 days after each switch (days 10, 30 and 40), only egg data after mating were included for comparisons between unmated and mated flies. A square root transformation was applied to egg production data to meet normality and homoscedasticity assumptions. All data are provided in supplementary information files.

## Results

### Essential nutrients decrease mortality rates, but do not mitigate previous aging

**Fig 1:**
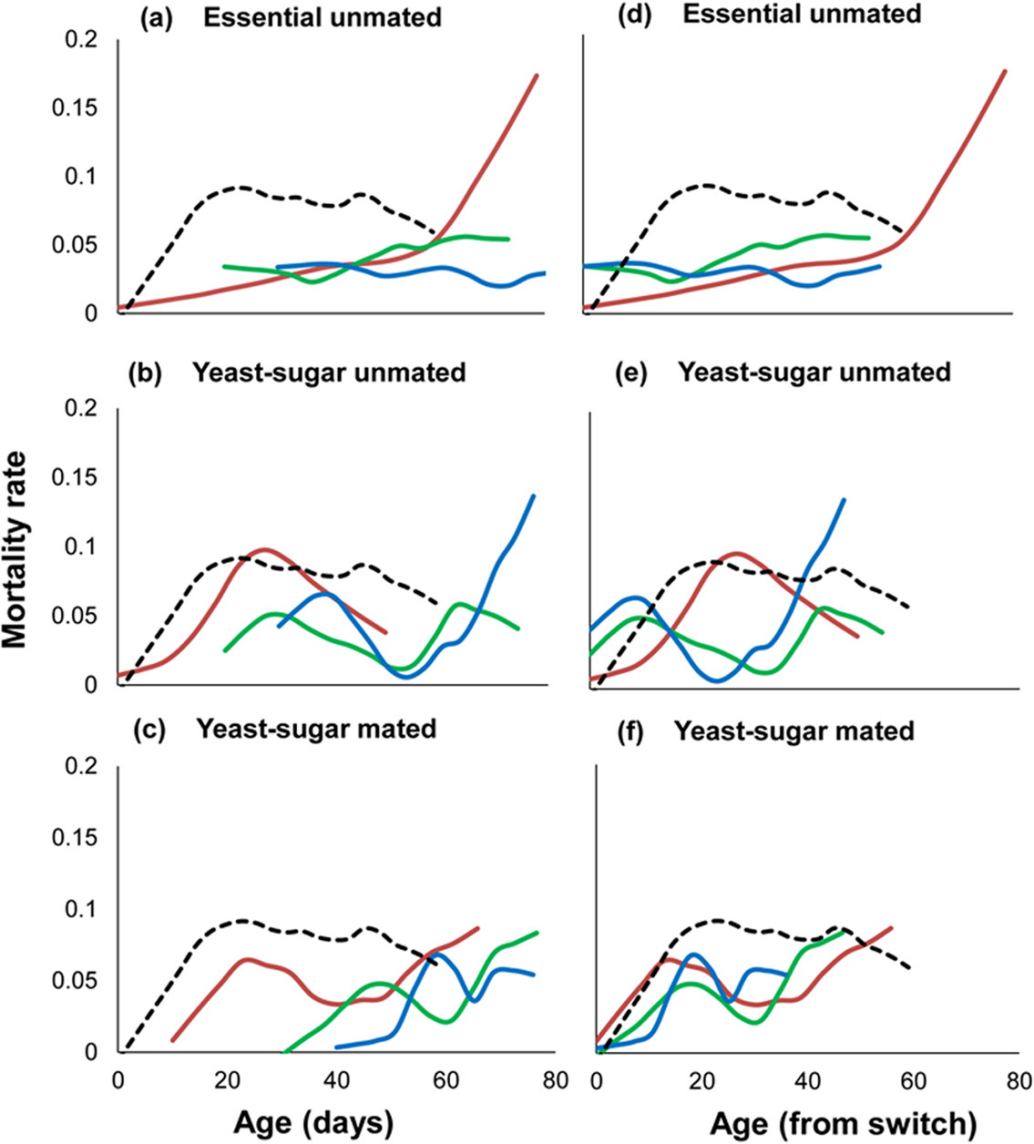
Smoothed mortality trajectories of Q-flies for each treatment. The left column (a, b and c) shows the mortality trajectories after Q-flies were switched from SUG to ESS or YS diet, respectively, in relation to time since emergence (Day 0 in red; Day 20 in green, Day 30 in blue). The mortality trajectory for flies maintained on SUG throughout is included in all figures (black, dashed line). The right column (d, e and f) shows the same mortality trajectories that are adjusted for the time from the diet switch (or mating for mated groups): For flies switched to the ESS or YS diet at 20 and 30 days old the x-axis has been re-scaled so that 0 represents the start of observations on the new diet. For YS-mated flies, the x-axis has been re-scaled so that 0 represents the start of observation from mating. Mortality rates were calculated until five individuals remained in each group.

Micronutrients in the ESS diet substantially increased longevity of female Q-flies with ESS-0 flies living over twice as long as SUG-0 flies (Table 1; 42.6 vs. 17.0 days, t_1323_ = 28.46, p < 0.001). This is also reflected in the shape of their mortality schedules (Fig 1A; χ^2^ = 124.41, p < 0.001); ESS-0 showed a gradual increase in mortality rate with age whereas SUG-0 flies showed a rapidly increasing mortality rate for the first 20 days and then remained fairly steady for the next 30 days (Fig 1A).

**Table 1:** Remaining life expectancy for each treatment at key time points. Life expectancy is defined here as the expected number of days remaining at day X. The switch day column refers to the day flies were switched from SUG to the corresponding diets. Mean remaining life expectancy is shown with ±SE and samples sizes in parentheses.

Treatment group	Switch day	Remaining Life Expectancy					
		0	10	20	30	40	50
Sugar	0	17.0±0.3	8.3±0.2	6.0±0.5	10.0±1.5	9.7±1.5	5.9±1.7
		(1028)	(927)	(307)	(51)	(23)	(11)
Essential	0	42.6±1.6	34.9±1.5	28.9±1.4	23.4±1.4	19.4±1.4	16.2±1.3
		(198)	(187)	(165)	(139)	(106)	(73)
	20			26.6±2.8	24.2±2.9	21.6±2.8	16.2±2.9
				(50)	(37)	(27)	(21)
	30				29.4±5.0	29.7±5.8	29.9±6.4
					(30)	(21)	(15)
Yeast-sugar unmated	0	23.8±1.4	17.8±1.2	11.0±1.3	8.5±1.9	10.6±2.7	8.8±2.8
		(100)	(82)	(65)	(31)	(11)	(5)
	20			19.6±2.7	19.4±3.9	26.7±4.7	24.9±4.6
				(63)	(37)	(19)	(14)
	30				16.6±2.5	21.3±4.2	26.6±4.0
					(57)	(25)	(14)
Yeast-sugar mated	0		23.1±2.0	15.5±2.1	16.8±2.8	19.9±2.7	13.0±2.5
			(73)	(64)	(33)	(17)	(14)
	20				29.8±2.8	21.2±2.8	17.2±2.9
					(32)	(30)	(22)
	30					30.2±3.9	21.4±3.9
						(21)	(20)

Switching flies from the SUG diet to the ESS diet later in life extended lifespan (Table 1; Fig 1A). At the point of the switch, ESS-20 flies had a higher remaining mean life expectancy of 26.6 days compared to only 6.0 days for SUG-0 flies (t_708_ = 9.45, p < 0.001). Similarly, at day 30, ESS-30 flies had a remaining life expectancy of 29.4 days compared to 10.0 days for SUG-0 flies.

Switching from SUG diet to ESS diet resulted in a complete reduction in age-specific mortality from the comparatively high level of SUG flies to the much lower levels of ESS-0 flies. Remaining life expectancies for ESS-20 after switching were remarkably similar to ESS-0 at the same chronological ages (Table 1). A comparison of mortality trajectories from day 20 between ESS-20 and ESS-0 revealed no significant difference in mortality rates (χ^2^ = 1.12, p = 0.40). ESS-30 flies also had a similar pattern of mortality rates dropping to those of ESS-0 flies for the first 20 days after switching (χ^2^ = 0.22, p = 0.74), but appear to have lower mortality rates thereafter (Fig 1A).

Although micronutrients extended lifespan, they did not mitigate previous aging effects. At the point of the diet switch, both ESS-20 and ESS-30 flies had shorter remaining life expectancy than the 42.6 day life expectancy of ESS-0 flies (Table 1; ESS-20 vs. ESS-0, t_275_ = 4.90, p < 0.001; ESS-30 vs. ESS-0, t_275_ = 3.68, p < 0.001). Furthermore, comparisons of mortality trajectories at the point of the diet switch (Fig 1D) found ESS-20 and ESS-30 both differed significantly from ESS-0 (χ^2^ = 26.53, p < 0.001; χ^2^ = 14.58, p < 0.001, respectively). ESS-20 flies consistently had higher mortality rates than the ESS-0 flies and ESS-30 flies had higher mortality rates than ESS-0 flies until about 30 days after switching (Fig 1D).

The ESS diet did not enable reproductive maturation. Post-mortem examination of ovarian development revealed that 25 out of 30 ESS flies had previtellogenic ovaries (Panel b in S3 Fig). Five ESS flies had more developed vitellogenic ovaries, but they still showed no evidence of mature egg formation. This was similar to SUG flies which all had previtellogenic ovaries (Panel a in S3 Fig). The majority of ESS and SUG flies produced no eggs during their lifetime (96.62% and 98.34% respectively). For those few ESS and SUG flies that laid eggs, lifetime egg production was very low (mean ± SD: 1.67±2.00 and 2.00±1.41 eggs respectively).

### Nutrients and reproductive maturation partially mitigate previous aging

Micronutrients did not mitigate previous aging effects. We next explored the effect of dietary yeast, which contains maturation-enabling protein in addition to the micronutrients of the ESS diet. Paralleling our approach with ESS diet, flies were switched from the nutrient-poor SUG diet to a nutrient-rich YS diet at 0, 20 and 30 days post-emergence.

The addition of yeast extended lifespan of virgin flies compared to flies on the SUG diet (Table 1; 23.5 vs. 17.0 days, t_1126_ = 6.01, p < 0.001). However, YS-0-U flies had a shorter life expectancy compared to the ESS-0 (Table 1; 23.5 vs. 42.6, respectively; t_296_ = 7.61, p<0.001), as a consequence of YS-0-U having consistently higher age-dependent mortality rates than ESS-0 (Fig 1A and 1B; Table 1; χ^2^ = 37.80, p < 0.001). Interestingly, the mortality trajectory for YS-0-U was similar in shape to the SUG diet with an increasing mortality rate for the first 20 days and then slowly decreasing thereafter (Fig 1B).

Switching flies from the SUG diet to the YS diet extended lifespan beyond same-aged flies on YS diet from emergence. At the time of the diet switch, both YS-20-U and YS-30-U flies had substantially higher remaining life expectancies compared to YS-0-U flies at the same chronological ages (Table 1; 19.6 vs. 11.0 days, t_126_ = 3.09, p = 0.003; 16.4 vs. 8.5 days, t_87_ = 2.17, p = 0.04 respectively). This extended lifespan is due to significantly lower mortality risk for YS-20-U (Fig 1B; χ^2^ = 22.33, p < 0.001) and YS-30-U (Fig 1B; χ^2^ = 75.20, p < 0.001) compared to YS-0-U.

The shape of the mortality schedules for flies switched to the YS diet at 20 or 30 days differed strongly from that of YS-0-U flies from the time flies were first exposed to YS diet (Fig 1E; χ^2^ = 21.81, p < 0.001; χ^2^ = 37.35, p < 0.001, respectively). YS-20-U and YS-30-U had similar shaped trajectories with mortality rates peaking 10 days after switching, then decreasing for 10 or 20 days (YS-30-U and YS-20-U, respectively) before increasing again (Fig 1E). In contrast, mortality rates for YS-0-U had the inverse pattern, peaking 30 days from first exposure to YS diet (Fig 1E).

The YS diet enabled reproductive maturation. All of the 33 YS flies examined retained eggs in their ovaries after they had died (Panel c in S3 Fig). In addition, virgin YS flies had substantial total egg production (mean ± SD: 130 ± 205 eggs).

### Nutrients, reproductive maturation and mating mitigate previous aging effects

The combined effects of acquiring nutrients and undergoing reproductive maturation did not induce the mitigation of previous aging effects that have been reported in medflies [7]. To investigate whether the additional intervention of mating triggered mitigation of prior aging, we considered female flies that were mated 10 days after being switched to the YS diet.

Mating increased remaining life expectancies from the time of mating (Table 1). YS-0-M, YS-20-M and YS-30-M had remaining lifespans of 23.1, 29.8 and 30.2 days respectively, whereas YS-0-U had 17.8 days (vs. YS-0-M; t_153_ = 2.19, p = 0.04), YS-20-U had 19.4 days (vs. YS-20-M; t_153_ = 3.38, p = 0.001) and YS-30-U had 21.3 days, respectively (vs. YS-30-M; t_153_ = 2.25, p = 0.04). YS-0-U and YS-0-M had similar mortality schedules for the first 15 days post-mating (Fig 1B and 1C; χ^2^ = 2.34, p = 0.19), diverging thereafter as the mortality rate of YS-0-U continued to increase for an additional 5 days and then remained consistently higher than YS-0-M (Fig 1B and 1C).

Lifespan extension from mating was caused by a reset of the mortality schedule to that of YS-0-M. Despite the differences in chronological ages, from the point of the mating intervention, mortality schedules of YS-20-M and YS-30-M did not differ significantly from YS-0-M (Fig 1F; χ^2^ = 0.13, p = 0.77; χ^2^ = 0.47, p = 0.62, respectively) or from each other (Fig 1F; χ^2^ = 0.004, p = 0.95). Each mating group had remarkably similar shaped mortality schedules, with mortality rates near zero almost immediately after mating, followed by increasing mortality rates until day 20 before stabilizing over the next 20 days (Fig 1F).

The proportion of flies mating at 10, 30 and 40 days was 0.77, 0.86 and 0.95 respectively. Mating facilitated the initiation of egg production; mated flies more often initiated egg laying than unmated flies (probability: 0.91 vs. 0.84, respectively; F_1,243_ = 4.62, p = 0.03).

**Fig 2:**
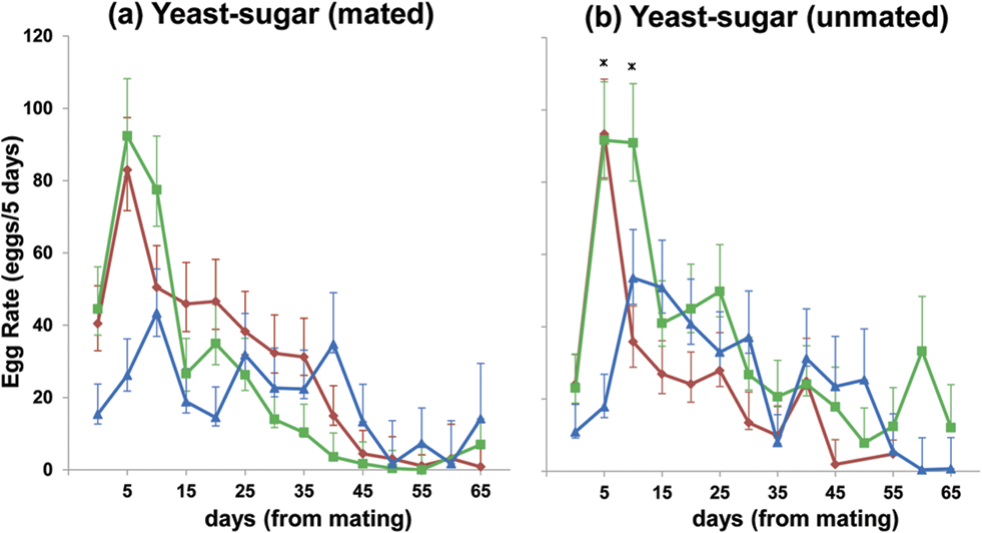
Egg production rates at 5-day intervals for YS 0, 20 and 30 mated (a) and unmated (b) flies that laid eggs. For YS 0, 20 and 30 mated and unmated flies, the x-axis has been rescaled so that day 0 represents day 10, 30 and 40 respectively (Day 10 in red; Day 30 in green, Day 40 in blue). Standard error bars are plotted at every 5-day interval and asterisk indicates significant difference (P<0.05) among treatments.

The age at which flies were switched to the YS diet affected egg production rates (Fig 2A and 2B, YS-M: F_25,482_ = 1.55, p = 0.04; YS-U: F_23,378_ = 2.20, p = 0.001 respectively). YS-0-M and YS-20-M flies had higher egg laying during the first 5 days after switching compared to YS-30-M flies (Fig 2A, F_2,375_ = 7.07, p = 0.002). YS-0-U and YS-20-U had higher egg laying for the first 5 days after switching compared to YS-30-U (Fig 2B, F_2,336_ = 10.32, p < 0.001). YS-20-U maintained a high rate of egg production up until the 10^th^ day after switching whereas egg production for YS-0-U was lowered (F_2,347_ = 4.79, p = 0.009). After this time, there were no differences in egg production rates for YS 0, 20 and 30 mated and unmated flies (Fig 2A and 2B).

## Discussion

Reproductively immature female Q-flies that are denied access to maturation-enabling protein exhibit an increasing mortality rate characteristic of aging but later mitigate this aging when provided maturation-enabling protein and then mated. This pattern closely resembles a previous report for medflies [7], suggesting this mechanism for minimizing aging at the onset of reproduction may be common in tephritid flies and possibly other anautogenous insects. Diet has previously been thought to be solely responsible for mitigation of aging in medflies switched from pre-reproductive waiting mode to a reproductive mode [7–9]. However, we found that flies that were provided maturation enabling protein and also mated were the only ones with mortality patterns that matched those of medflies [7]. Hence mating is an important trigger for mitigation of aging. Despite YS-20-M and YS-30-M flies being 30 and 40 days old when first mated, their mortality rates dropped rapidly to zero immediately after mating and thereafter matched that of YS-10-M flies when they were first mated (Fig 1F). Hence, amount of time in the pre-reproductive adult stage had very little bearing on subsequent lifespan post-mating. In comparison, the essential and yeast-sugar unmated flies had mortality rates that continued to increase with age at the same time points and full life expectancy was not recovered. This distinction between mated versus unmated YS and ESS flies, demonstrates that mating is a key trigger for mitigation of previous aging.

Mating in flies triggers a cascade of physiological changes that support reproduction. Mating in *Drosophila melanogaster* females activates a diverse array of genes involved in biological processes associated with lifespan, such as metabolism, immune defense, and endocrine signaling pathways [14, 22, 23]. Mating increases metabolic rate in *D*. *simulans* females [24], activates several antioxidants in *D*. *melanogaster* females that may have protective effects [14], and up-regulates many genes encoding for antimicrobial peptides of the humoral immune system. Furthermore, mating increases juvenile hormone biosynthesis in *D*. *melanogaster* [25], which appears closely linked to lifespan modulation [9, 26, 27]. Although far less studied, mating also triggers physiological and behavioural changes in tephritid flies that generally resemble those reported in *Drosophila*. In both medflies and Q-flies, mating promotes oviposition and also sexual inhibition, an effect mediated by accessory gland fluids in the ejaculate [28, 29]. Given their known role in mediation of female reproduction, male accessory gland fluids are prime candidates as a trigger for mitigation of previous aging in both of these tephritid flies.

Mitigation of aging during ontogenic transitions also seems to occur in adult honeybees when switched from the foraging adult stage back to the nursing stage (reviewed in [12, 13]). Reverted nurse bees have restored brain [30] and immune function [31], matching levels of much younger nurse bees. Furthermore, though bees age during the nursing stage, the ontogenic shift to the foraging stage appears to reverse previous aging as the mortality trajectory of new foragers appears independent of the chronological age of the bee [12]. Vitogellenin and juvenile hormone have been suggested as important endocrine mediators of this onotogenetic transition in honeybees [12]. These hormones are also vital to reproductive maturation and egg production in flies and likely play a role in the mitigation of aging reported in the present study [32, 33].

Though lifespan was renewed in mated Q-flies, egg production rates for mated flies switched at day 30 were significantly reduced. Similar results have been reported for medflies [7], as well as *D*. *melanogaster* that had been induced into reproductive diapause for several weeks [9]. Therefore, the reproductive system appears to age continually during the pre-reproductive and reproductive stages and the ontogenic switch to the reproductive stage does not, at least not completely, reverse reproductive aging.

Micronutrients do not alter age-dependent mortality rates in Q-flies, but they do reduce age-independent mortality risk [8, 34, 35]. Switching SUG Q-flies to the ESS diet resulted in flies rapidly adopting the mortality schedule of ESS flies of the same chronological age. As the mortality schedule of flies in the SUG group likely in part reflects malnutrition (paucity of vitamins, minerals, sterols and amino acids [18]), our results suggest that flies receiving the SUG diet were aging at a rate similar to flies receiving the ESS diet, leading to similar age-dependent mortality rates, but were also experiencing much higher levels of age-independent mortality risk associated with malnutrition.

In contrast to the clear patterns for flies switched to the essential diet and for the mated flies (YS-fed), the mortality patterns for flies provided a maturation-enabling diet later in life but not mated (YS-20-U and YS-30-U) were less clear. These flies exhibited a drop in mortality rate similar to that of flies switched to the essential diet. However, unlike flies switched to the essential diet, the (YS-fed) unmated flies exhibited a dip in mortality, suggesting some mitigation of previous aging. The apparent partial effects of diet in the absence of mating may reflect heterogeneity in the extent to which the maturation-enabling diet alone allowed individual flies to mitigate previous aging. The partial effects of diet alone on population aging schedule may arise because individual flies vary either in the probability or extent to which they transition to the reproductive stage. The full response seen in mated females may reflect the efficacy of mating as a trigger for full transition to the reproductive stage by the large majority of individual flies.

Aging theory predicts that insects should be under strong selection pressure to minimize aging prior to reproduction in order to preserve reproductive lifespan. During pre-reproductive adulthood, female Q-flies and medflies [7] have increasing mortality curves characteristic of aging, suggesting that at least for some species it may not be possible or beneficial to completely prevent pre-reproductive aging. Instead, these flies rely on a strategy of mitigating effects of pre-reproductive aging on mortality rates during the ontogenic transition to reproductive activity. Our results consistently showed that mortality rates and patterns after mating were highly similar for flies mated at different ages, regardless of the amount of time spent in the pre-reproductive adult stage. These results closely match trends reported previously for mated medflies [7], but with an experimental design that has allowed us to disambiguate the effects of diet and mating. We conclude that mating is a key trigger for mitigation of aging that allows Q-flies to synchronize reproduction and mortality schedules. The next step is to identify what physiological mechanisms associated with mating are responsible for mitigating previous aging effects.

## Supporting Information

S1 Table Ingredients for each experimental diet to which 1 L of deionized water was added. (DOCX)

S1 Fig Schematic diagram showing adult mortality trajectories that would indicate a) reproductive diapause and b) mitigation of aging. Different colored lines indicate different durations of the pre-reproductive adult stage (red = species that emerge as sexually mature adults; green and blue = species that enter reproductive diapause for different lengths). Vertical reference lines indicate the onset of reproduction. For species that enter reproductive diapause, aging does not start until the species exits reproductive diapause (e.g., Drosophila [1]). For species that mitigate previous aging, the aging process starts immediately during the adult stage but then is “reset” when sexual maturity is reached (e.g., medflies [7]).
(TIF)

S2 Fig Diagram showing how each treatment group was established after emergence. For a subset of Q-flies switched to the YS diet, this also included the opportunity to mate 10–11 days later.
(TIF)

S3 Fig Typical ovarian dissections from flies switched to or permanently fed a) SUG, b) ESS and c) YS diet. Ovarian development was scored post-mortem on a scale of 1 to 5 following [20] and whether eggs were present.
(TIF)
