# Supplementary material for: Mating Reverses Actuarial Aging in Female Queensland Fruit Flies
Source: PLoS One. 2015 Jul 6;10(7):e0132486. doi: 10.1371/journal.pone.0132486 (PMC4492602; doi:10.1371/journal.pone.0132486)
Supplement: S1 Table — (DOCX) [file pone.0132486.s001.docx]

**S1 Table.** Ingredients for each experimental diet to which 1 L of deionized water was added

| Ingredients | Diet (g) | | | Producer (Cat. #) |
| --- | --- | --- | --- | --- |
|  | Sugar | Essential | Yeast-sugar |  |
| Sucrose | 300 | 300 | 225 | Sigma (84100) |
| Yeast hydrolysate |  |  | 75 | MPBio (103304) |
| Cholesterol |  | 1 |  | Sigma (C3045) |
| Vanderzant Vitamin Mixture |  | 1 |  | Sigma (V1007) |
| Wesson Salt Mixture |  | 2 |  | MPBio (0290285102) |
| Agar USP grade | 15 | 15 | 15 | MPBio (0210026280) |
| Methyl 4-hydroxybenzoate | 2 | 2 | 2 | Sigma (H3647) |
