# Supplementary figures and images for: Mating Reverses Actuarial Aging in Female Queensland Fruit Flies
Source: PLoS One. 2015 Jul 6;10(7):e0132486. doi: 10.1371/journal.pone.0132486 (PMC4492602; doi:10.1371/journal.pone.0132486)

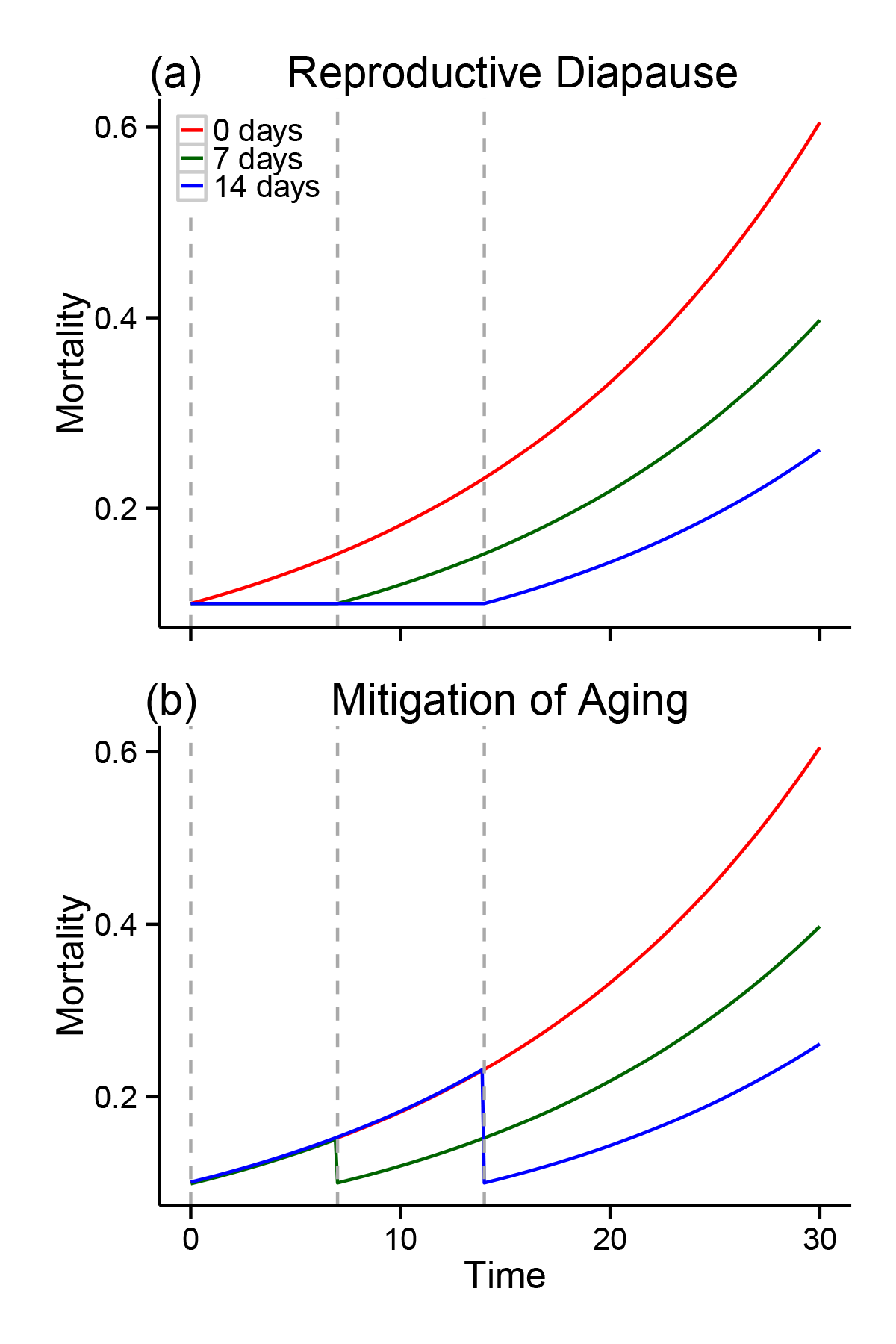

Supplement: S1 Fig — Different colored lines indicate different durations of the pre-reproductive adult stage (red = species that emerge as sexually mature adults; green and blue = species that enter reproductive diapause for different lengths). Vertical reference lines indicate the onset of reproduction. For species that enter reproductive diapause, aging does not start until the species exits reproductive diapause (e.g., Drosophila [1]). For species that mitigate previous aging, the aging process starts immediately during the adult stage but then is “reset” when sexual maturity is reached (e.g., medflies [7]). (TIF) [file pone.0132486.s002.tif]

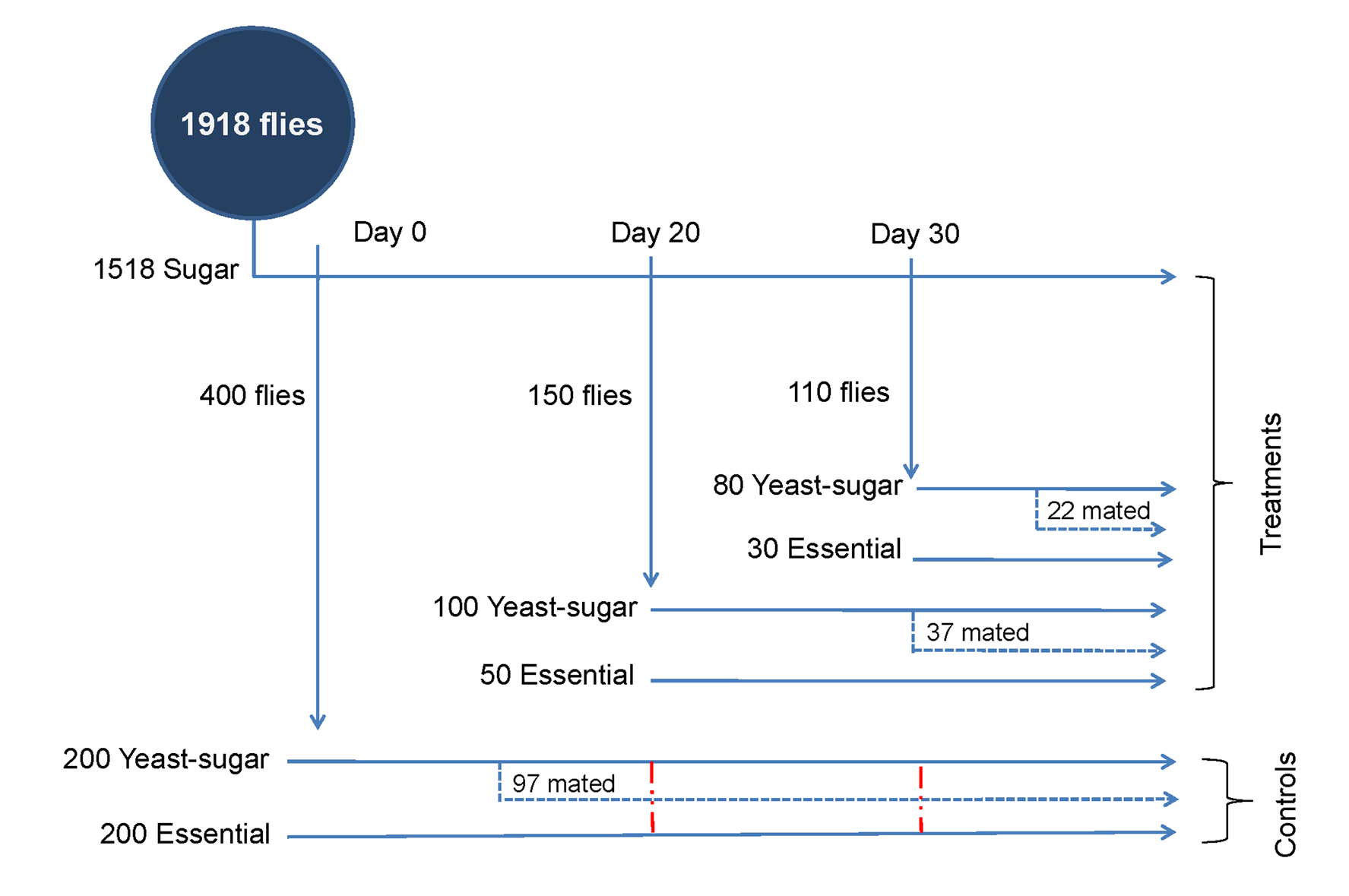

Supplement: S2 Fig — For a subset of Q-flies switched to the YS diet, this also included the opportunity to mate 10–11 days later. (TIF) [file pone.0132486.s003.tif]

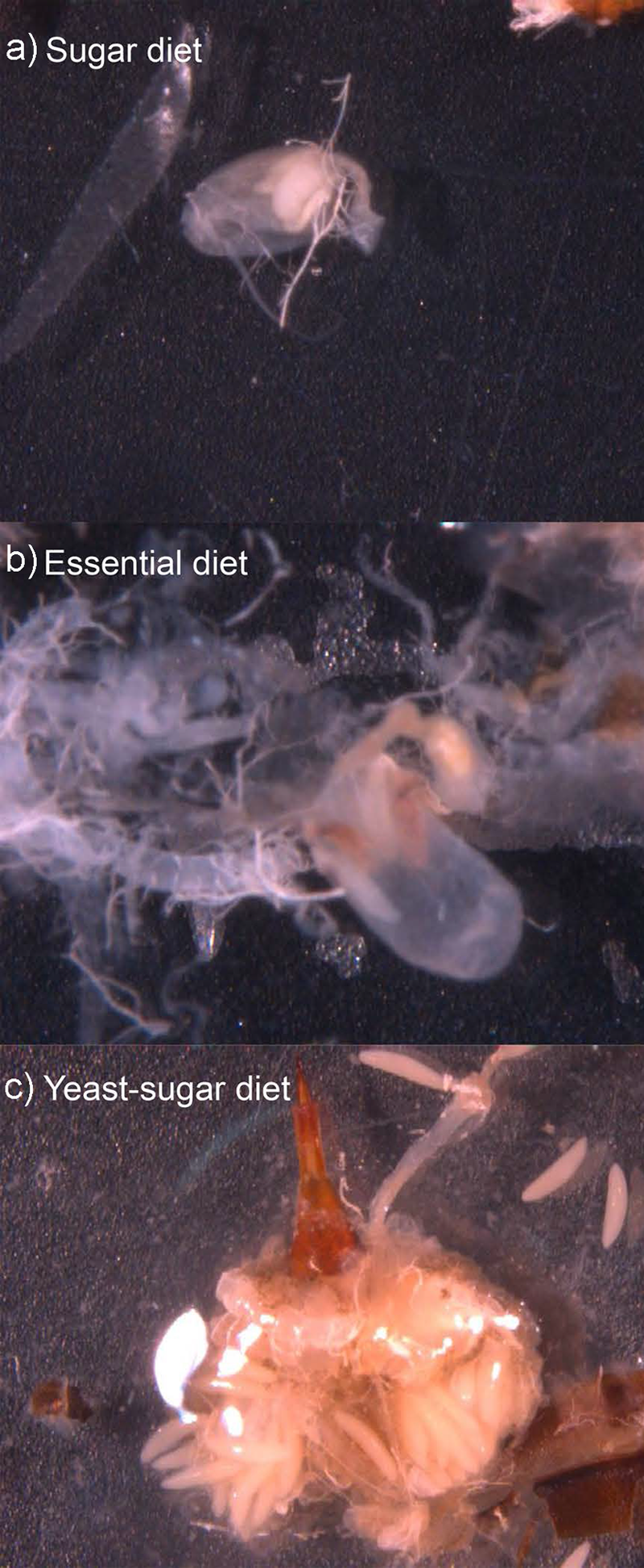

Supplement: S3 Fig — Ovarian development was scored post-mortem on a scale of 1 to 5 following [20] and whether eggs were present. (TIF) [file pone.0132486.s004.tif]
